# Atrial delayed enhancement is associated with the severity of diastolic dysfunction in cardiac amyloidosis

**DOI:** 10.1186/1532-429X-15-S1-P143

**Published:** 2013-01-30

**Authors:** Eduardo Pozo, Jose M Castellano, Tara Naib, Rajiv Deochand, Anubhav Kanwar, Ines Garcia-Lunar, Pablo Pazos, Jill Kalman, Valentin Fuster, Javier Sanz

**Affiliations:** 1 Cardiology, Mount Sinai School of Medicine, New York City, NY, USA

## Background

Interstitial deposition of amyloid fibrils in the heart causes thickening of atrial and ventricular walls. Although the presence of abnormal late gadolinium enhancement (LGE) in ventricular myocardium is well described, the significance of atrial LGE is less clear.

The aim of this study is to describe the prevalence of atrial LGE in cardiac amyloidosis and to investigate potential associations with disease severity.

## Methods

We retrospectively reviewed consecutive patients referred for CMR due to clinically suspected cardiac amyloidosis. The final diagnosis of cardiac amyloidosis was established in the presence of a positive cardiac biopsy and/or a typical pattern of diffuse, predominantly subendocardial left ventricular (LV) LGE. Clinical data (including EKG, pulmonary capillary wedge pressure from cardiac catheterization and brain natriuretic peptide [BNP] serum levels) and subsequent events (death or hospitalization) were recorded. Indexed LV and right ventricular (RV) volumes and ejection fractions, LV mass, LV basal anteroseptal and inferolateral end-diastolic wall thicknesses, and left atrial dimensions were determined from standard cine CMR images. In cardiac amyloid patients, the septal E/E' ratio, as an index of LV filling pressures, and diastolic function were determined from Doppler echocardiography. Diastolic function was classified as grades 0 (normal), I (impaired relaxation pattern), II (pseudonormal pattern), and III (restrictive pattern).

## Results

We included 125 patients (85 males [68%], age 63±13 years) referred for CMR (59 (47%) at 1.5, 66 (53%) at 3.0 Tesla), of which 51 (40.8%) were diagnosed of cardiac amyloidosis. The prevalence of atrial LGE was markedly higher in those with a diagnosis of cardiac amyloid (86.3% vs 13.5%, p<0.001). Including all 125 patients, atrial LGE was independently associated with the diagnosis of cardiac amyloid in a multivariate analysis including all CMR variables (odds ratio [OR] =26.9, 95% confidence intervals 8.1-88.7, p<0.001).

**Figure 1:**
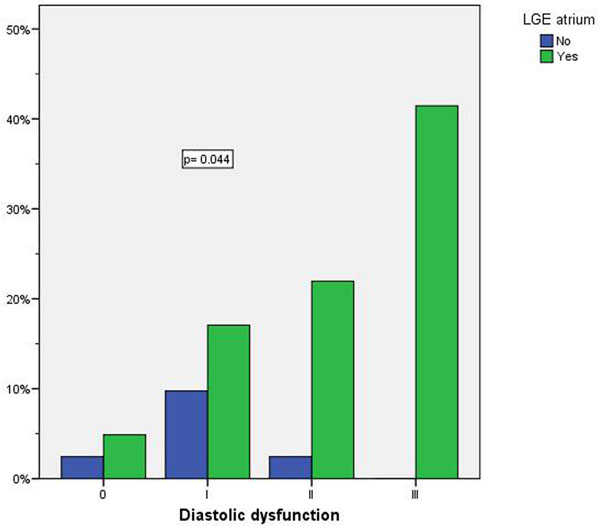
Association between diastolic dysfunction severity and presence of atrial LGE.

In the group of patients with cardiac amyloidosis, the presence of atrial LGE was associated with age (68 ± 12 vs 58 ± 15 years in those with and without LGE, respectively; p=0.041), and severity of diastolic dysfunction (r=0.422, p=0.006, Figure 1). There were no significant associations with prevalence of atrial fibrillation, ventricular or left atrial measurements, pulmonary capillary wedge pressure, BNP, or follow-up events.

## Conclusions

The presence of atrial LGE is independently associated with the diagnosis of cardiac amyloid. In patients with cardiac amyloidosis, atrial LGE is related to age and the severity of diastolic dysfunction.

## Funding

No funding sources have to be declared

